# Knowledge and practices of primary care physicians or general practitioners treating post-infectious Irritable Bowel Syndrome

**DOI:** 10.1186/s12876-020-01305-z

**Published:** 2020-05-25

**Authors:** Erika Austhof, Kenzie Schaefer, Jaime Faulkner, Laura Bach, Mark Riddle, Kristen Pogreba-Brown

**Affiliations:** 1 Department of Epidemiology & Biostatistics, University of Arizona, Mel and Enid Zuckerman College of Public Health, 1295 N Martin Ave, PO Box 245211, Tucson, 85721 AZ USA; 2 College of Medicine, University of Arizona, 1501 N Campbell Ave, PO Box 245017, Tucson, 85724 AZ USA; 3 Division of Gastroenterology and Hepatology, College of Medicine, University of Arizona, 1501 N Campbell Ave, PO Box 245028, Tucson, 85724 AZ USA; 4 School of Medicine, University of Nevada Reno, 1664 N Virginia Street, Reno, 89557 NV USA

**Keywords:** Irritable bowel syndrome, General physician, Treatment, Knowledge, Survey

## Abstract

**Background:**

Post-infectious Irritable Bowel Syndrome (PI-IBS) is a functional bowel disorder which has significant impacts to a patient’s quality of life. No IBS-specific biomarker or treatment regimen for PI-IBS currently exists, therefore understanding practice patterns and variance is of interest.

**Methods:**

This online survey of primary care physicians and general practitioners in the USA aimed to understand the knowledge and treatment of PI-IBS within the physician’s current practice. Summary statistics are provided with a commentary on implications for practices and treatment of PI-IBS.

**Results:**

Most physician survey respondents (*n* = 50) were aware of PI-IBS, but less than half discussed this condition as a possible outcome in their patients with a recent gastrointestinal infection. Most physicians indicated that they would treat the patients themselves with a focus on managing IBS through different treatment modalities based on severity. Treatment for PI-IBS followed IBS recommendations, but most physicians also prescribed a probiotic for therapy. Physicians estimated that 4 out of 10 patients who develop PI-IBS will have life-long symptoms and described significant impacts to their patient’s quality of life. Additionally, physicians estimated a significant financial burden for PI-IBS patients, ranging from $100–1000 (USD) over the course of their illness. Most physicians agreed that they would use a risk score to predict the probability of their patients developing PI-IBS, if available.

**Conclusions:**

While this survey is limited due to sample size, physician knowledge and treatment of PI-IBS was consistent across respondents. Overall, the physicians identified significant impacts to patient’s quality of life due to PI-IBS.

## Background

Post-infectious Irritable Bowel Syndrome (PI-IBS) is a functional bowel disorder in which recurrent abdominal pain is associated with defecation or a change in bowel habits after “an episode of acute gastroenteritis in individuals who did not have IBS before the infection” [1]. Recent meta-analyses [2] found more than 10% of patients who had infectious enteritis within the previous 12 months had a 4-fold higher risk of developing IBS compared to patients without a previous infection. Additionally, 6–17% of patients believe their IBS began after an infection [3, 4]. Since no IBS specific biomarkers exist, primary care physicians (PCPs) and general practitioners (GPs) rely on evaluation of patient symptoms and IBS is often diagnosed only after other conditions are excluded [5, 6]. While formal diagnostic tools including the Rome IV and Manning criteria are available, a minority of PCPs are aware of these tools (2–36%) and even less (0–21%) use them [7]. Despite being a non-life threatening illness, IBS can greatly affect a patient’s quality of life and is associated with high rates of depression. Therefore, it is important physicians recognize and treat IBS based on evidence-based therapies [7]. Many studies have assessed physician’s evaluation and treatment of IBS around the world, with two in the US [8, 9] but none have focused on PI-IBS. The objective of this pilot study is to assess physician knowledge and treatment specific to PI-IBS within their practice.

## Methods

Following a literature review, the survey was drafted and reviewed by members of a workgroup that is focused on the chronic outcomes associated with foodborne illnesses. The workgroup consists of experts in epidemiology, gastroenterology, foodborne diseases and government regulation. With the goal of focusing the survey on PI-IBS, questions were edited and tailored to fit a non-gastroenterologist physician audience. The survey questions were piloted with a medical resident (JF), and a gastroenterology fellow (LB) to develop face validity. Any questions deemed not effective were re-worded and re-piloted until the intended aims of the question were reached.

The survey included four major sections: 1) basic demographics (no personal identifiers were collected) and knowledge of chronic outcomes from common foodborne diseases; 2) knowledge and practices related to diagnoses, treatment steps of PI-IBS, and prescription of medications; 3) knowledge and practices of assessing quality of life; and 4) attitudes around utilizing a hypothetical risk score and modes of use. By knowledge we mean the facts, information, and skills acquired by the physician through their experiences in clinic or through education. To reduce respondent confusion we framed some questions on patient interactions using the general IBS term because 1) there is not a widely used diagnostic test to distinguish PI-IBS and general IBS and 2) general IBS and PI-IBS do not currently have different treatment recommendations. The online survey included 40 questions, was available in English, and was estimated to take 11 min.

Our team worked with Qualtrics (Provo, UT) an online survey company to recruit a sample of 50 U.S. physicians. We did not provide qualifications on age, race, or gender for sampling in this survey. Physicians were screened on three questions: 1) “Do you personally see and treat patients for Irritable Bowel Syndrome (IBS) in your practice?” 2) “Do you personally see and treat patients for foodborne illness in your practice?” and 3) “Which best describes your current occupation?” Survey respondents were required to answer “Yes” to the first two questions and select “Primary Care Physician or General Practitioner” in order to participate in the survey. The subjects were not screened on the type of residency or prior training completed, only that they currently practice as a PCP or GP. The survey was launched online July 11, 2019 and remained open until July 16, 2019.

All data were analyzed using embedded analysis programming within Qualtrics (Provo, UT) and Stata 14.0 (STATA Corp, College Station, TX). Sample size (*n* = 50) for this pilot study was based on availability of resources to complete the survey. If responses to a question followed a normal distribution, mean proportion with standard deviation (SD) and range are provided. If responses to a question followed a non-normal distribution, the median proportion and interquartile range (IQR) are provided. There was no missing data in the dataset.

The University of Arizona Institutional Review Board reviewed this project and determined that the research is considered exempt human subjects research. Qualtrics provided an incentive (~$100 USD) for each physician to complete the survey.

## Results

The survey took an average of 9.9 min to complete (Range: 3.4–38.5 min). The majority of survey respondents were male, had an MD, were practicing for 10 years or more, and resided in the Midwest or Northeast regions of the USA (Table 1). As part of eligibility requirements to take the survey, all respondents (*n* = 50) affirmed that they were currently a PCP or GP and they personally treat and see patients for foodborne illness and IBS within their current practice. Two participants indicated that their residency specialty was Anesthesiology and Pathology, respectively; however they did confirm “yes” to both of the eligibility questions and their answers were not outliers from the other 48 participants.

**Table 1:** Demographics of primary care physician and general practitioner survey respondents (*N* = 50), July, 2019

Category	N (%)
Gender	
Male	35 (70)
Female	15 (30)
Medical Degree	
MD	43 (86)
DO	7 (14)
Residency Specialty	
Family Practice	25 (50)
Internal Medicine	23 (46)
Other (Anesthesiology, Pathology)	2 (4)
Length of Practice (years)	
1–4 years	3 (6)
5–10 years	6 (12)
10 years or more	41 (82)
Region of the United States (state abbreviation)	
West (WA, OR, CA, CO, UT, HI)	8 (16)
Midwest (KS, MO, IA, WI, IN, IL, MI)	16 (32)
Southwest (AZ, TX)	4 (8)
Southeast (FL, GA, TN, KY, WV)	9 (18)
Northeast (CT, MA, MD, NJ, NY, PA)	13 (26)

Most physicians saw multiple patients with an acute foodborne illness each month (62%), multiple within a week (22%), or multiple within a day (10%). Almost all physicians (88%) noted that they are aware of specific foodborne pathogens that can cause functional gastrointestinal disorders including: *E. coli* (*n* = 33, 66%), *Salmonella* (*n* = 30, 60%), *Campylobacter* (*n* = 28, 56%), *Giardia* (*n* = 27, 54%), *Cryptosporidium* (*n* = 24, 48%), or all listed pathogens (*n* = 22, 45%; other pathogens listed include *Shigella*, *Listeria*, *Yersinia*, *Cyclospora*, and *Norovirus*). Among a list of gastrointestinal disorders, the majority of physicians (*n* = 33, 66%) noted IBS, followed by chronic diarrhea (*n* = 29, 58%), and dyspepsia (*n* = 22, 45%) as gastrointestinal conditions most commonly linked to foodborne illness or which could have resulted from infection with a foodborne pathogen. Forty-eight percent responded they often or always discuss possible long-term outcomes of chronic conditions that might occur after foodborne or gastrointestinal illness.

When considering diagnoses for suspect PI-IBS patients, 40% of physicians review the patients’ history for a previous gastrointestinal illness and the majority look up to 1 year prior (54%). Almost all physicians (90%) review previous diagnostic lab results or ask their patients if a sample tested positive for a specific pathogen. Physicians estimated that 42% (SD = 22.9, range: 5–94%) of their patients diagnosed with IBS report a gastrointestinal illness prior to their diagnosis. The majority of physicians (74%) agreed that knowing a patient had a previously diagnosed foodborne infection would change the way they approached treatment of their IBS patients. As their first diagnostic step after a patient developed PI-IBS, 32% of physicians ordered a follow-up stool culture (32%) or other laboratory test (16%). For physicians who selected “I would order a follow-up stool sample” (*n* = 13), their reasons included wanting to rule out ongoing/persistent infection (*n* = 11) or parasitic infection (*n* = 2).

In general, the physicians’ first treatment step for PI-IBS patients was prescriptions for probiotics (66%) or antibiotics (28%). No physicians prescribe antidepressants as their first treatment step, and 46% made recommendations for dietary changes. For a patient with PI-IBS, most physicians would treat the patient themselves (54%) and manage the illness through treatment modality modification depending on severity of the PI-IBS (44%). Only one PCP noted they would refer the patient to a specialist. Of the 20 physicians who provided additional comment, physicians estimated 16.5% on average (range: 5–30%) of their IBS patients are severe (no specific definition). PI-IBS was deemed as “severe” when physicians saw the patient more often, referred them to a gastroenterologist for additional management, or considered other modalities such as referring to a psychiatrist or trying a different antibiotic.

Physicians prescribed laxatives for general IBS patients with the constipation sub-type and anti-diarrheal medications or gut antispasmodics for general IBS patients with the diarrhea sub-type most often (Table 2). Physicians believed that patients stayed on these medications for less than 6 months (36%) or from 6 months to 1 year (36%). Physicians typically see their IBS patients every 3 months (84%) and most estimate less than 10% of their patients (median: 8.5%, IQR: 4–19%) are hospitalized one or more times per year for their IBS.

**Table 2:** List and count of common medications (*N* = 123) prescribed by primary care physician and general practitioner survey respondents (*N* = 50), July, 2019

Medication	N
Prescription Anti-Constipation (Lubiprostone, Linaclotide)	30
OTC Anti-constipation (Polyethylene glycol, Docusate, Senna)	8
Antispasmodic (Dicyclomine, Hyoscyamine)	18
Prescription Anti-diarrheal (alosetron hydrochloride, eluxadoline, loperamide hydrocholride)	9
OTC Anti-diarrheal (loperamide, constipating agents)	13
Antibiotics (rifaximin)	9
Fiber	9
Serotonin or norepinephrine reuptake inhibitors	6
Tri-cyclic Antidepressants (amitriptyline, nortriptyline)	5
Probiotics	4
Aminosalicylates (mesalamine)	1
Other (acid reducer, motility agents, stool softeners)	11
**Total**	**123**

Physicians were split on whether they discuss psychological health impacts resulting from their patient's PI-IBS (never 4%, sometimes 20%, half the time 16%, most of the time 34%, always 26%). More than half the physicians ask about impacts to their patients’ quality of life (most 34%, always 36%) and provided in-depth examples of those impacts. Physicians noted avoidance of social events, impacts to work life, and impacts to their mental health and relationships most often; quotes from survey respondents are shared in (Table 3). Most physicians estimate that their IBS patients lose less than 10 h per week out of a 40-h work week (median: 8 h, IQR: 3–18 h) and less than 5% (median: 5%, IQR: 1–16%) go on permanent disability due to their IBS. Twenty percent of physicians believe IBS resolves after 1–5 years, another 22% believe IBS resolves after 6–12 months, one physician believed IBS resolves in less than 6 months, while 42% (SD = 25.45, range 5–100%) believe IBS patients never resolve their IBS symptoms. Most physicians believed IBS patients spend $100–500 USD (40%) or $500–1000 USD (38%) in out-of-pocket medical expenses over the course of their illness.

**Table 3:** Impacts to IBS patient Quality of Life provided by general physician survey respondents (*N* = 50), July, 2019

Dimension	Quality of Life Impact
Social	• “Unable to socialize due to fear of unexpected diarrhea” • “Frequent trips to the bathroom and limiting their social life” • “Afraid to travel, afraid to go out with friends or family, afraid to go to public events” • “Can’t work, can’t go on trips, have to know where all bathrooms are, can’t do activities outdoors” • “Reduced social activities due to bathroom needs”
Employment	• “Loss of employment/number of sick days” • “Not being able to hold down a job” • “Missing work, or unable to keep appointments due to symptoms of IBS” • “They plan a Saturday to take laxatives and stay by the toilet or they know where every bathroom is on the drive from one place to the next; they don’t want to leave the house or go out to eat”
Mental Health	• “Hesitation to participate in social functions, decreased confidence” • “Anxiety. Depression” • “Afraid to leave home” • “Anxiety, fear of eating out, fear of dining, embarrassment” • “Constant awareness of where a bathroom is” • “Absence from work, Social Withdrawal, Social Isolation.”

Finally, 60% of physicians agreed that they would use a user-friendly risk score that would help them predict whether a patient would develop PI-IBS. Among the formats available, physicians said they would be most likely to use a printout with a risk score (36%), a website with a risk score calculator (30%), or a phone application with a risk score calculator and informational materials for their patient (28%).

## Discussion

This study is the first to identify interesting and relevant descriptions to PCP and GP knowledge and treatment of PI-IBS. Overall, we found a wide range of understanding of which common foodborne pathogens can lead to functional gastrointestinal disorders such as PI-IBS. Awareness of PI-IBS as a phenomenon is present in a majority, but less than half of the physicians surveyed would discuss this as a possible outcome in their infectious GI patients. Given the known frequency of PI-IBS after GI infections, universal discussion with the patient of chronic consequences may be important.

The estimate that 42% of new IBS diagnoses are suspected to have a post-infectious etiology is higher than has been previously reported [2]. It may be that as awareness of PI-IBS among physicians has grown, more providers are taking detailed histories of antecedent acute GI illness and identifying a higher proportion of patients with such a trigger. Alternatively, the sample selection may have been biased or the survey design led respondents to inflate estimates of an infectious trigger. While most physicians agreed that knowing a patient had a previously diagnosed foodborne infection would change the way they approached treatment of their patients IBS, we did not ask how it would change their approach or how often they find a result. Given that there are no targeted therapies for PI-IBS at this time, we expect treatment should not change from IBS recommendations. However, it is possible that the time from diagnosis to treatment for the patients IBS may be shorter as there would be less diagnostic work-up to rule out potential etiologies.

In our survey, physicians frequently prescribed a probiotic for therapy. This management approach is not consistent with current guidelines for practice. The American College of Gastroenterology guidelines give the following recommendation, “We suggest probiotics, taken as a group, to improve global symptoms, as well as bloating and flatulence in IBS patients. (Recommendation: weak; Quality of evidence: low)”. Similarly, the Canadian Association of Gastroenterology states, “We suggest offering IBS patients probiotics to improve IBS symptoms (GRADE: Conditional recommendation, low-quality evidence)” [10]. While these recommendations don’t discourage use of probiotics, we are unsure why there is an observed practice variation between current practice guidelines and reported practice of this sample population. It is possible that patients are driving demand for probiotics based on their review and awareness of medical information from online sources and marketing. Most probiotics are OTC and, thus, it could be that while physicians did not recall them as often in the list of common medications they prescribe (as seen in our results for Table 2) physicians are supportive in recommending OTC probiotics for therapy when patients ask. In any case, given the lack of evidence for probiotics in the effectiveness of IBS therapy [11] physicians may not be fully helping their patients to avoid the excess costs (most often out of pocket) associated with therapies that lack proven effectiveness.

Interestingly, the estimate that roughly 4 out of 10 patients who develop PI-IBS will continue to have symptoms throughout their life is an important observation. Reported literature have only followed up subjects for up to 8 years [12]. To determine the prevalence of PI-IBS among Walkerton Health Study participants, 28.3% (*n* = 210) of patients enrolled into the PI-IBS cohort reported the condition after 2–3 years. At the 8-year follow-up, the overall prevalence within the cohort dropped to 15.4%, but over 50% of those diagnosed at year 2–3 were still symptomatic at year 8. These findings are consistent with the estimates given in this survey. While there exist no studies that have estimated the lifelong impact of PI-IBS, for some, IBS remains a chronic condition.

In addition to management, this survey focused on providers’ assessment of the functional impact associated with PI-IBS. While there may be cognitive bias (e.g. physicians will often focus on the more severe patients as representative of the general population of patients), the sample respondents describe significant impacts for the PI-IBS patient populations they manage. Because quantitative techniques may not be feasible in measuring symptom management, quality of life assessments remain an imperative aspect in follow up care among this patient population. In this sense, physicians will be able to gain a better understanding of treatment efficacy based on quality of life discussions prior to and during the treatment course. Buono et al. [13] found that patients with IBS-D reported significantly lower health-related quality of life than controls; these findings are consistent with our response from physicians. For example, physicians from our survey estimated an average loss of 10 h per week in their patients suffering from IBS, and also noted multiple employment-based impacts on quality of life due to their symptoms (Table 3). Buono et al. [14] found IBS-D patients had a 20.7% productivity loss compared to controls and had a significant daily activity impairment (29.5%) whereas Tack et al. [15] found those with IBS-C had a 27.7–51.5% productivity loss and an overall daily activity impairment from 36.4–56.8%. By discussing baseline symptoms and impacts on quality of life and subsequently comparing original answers to those after starting the treatment course, physicians can more easily recognize whether treatment is alleviating or aggregating symptoms [16].

In addition to an assessment of psychological and social impacts on patient life, it is important to address the economic burden incurred due to this chronic disease. The physicians in our survey estimated the average patient incurs $100–500 USD (40%) or $500–1000 USD (38%) in out-of-pocket expenses during the course of their illness which can be a significant financial burden for some. Buono et al. [14] demonstrated a significant economic burden associated with IBS-D in a US population, however they did not estimate out of pocket medical expenses in this population. These estimates could also vary widely given a patients insurance coverage, even if only considering the range of coverage for the recommended medications the survey participants noted. Together, these descriptions describe PI-IBS as a condition with significant economic and societal burden for which improved diagnostics, preventive strategies and effective treatments are sorely needed.

While no physicians initially prescribed antidepressant medication, 9% of the medications listed are nerve pain medication or antidepressants, SSRIs, or SNRIs. The American College of Gastroenterology recommends tricyclic anti-depressants (TCAs) for overall symptom improvement in IBS patients with a strong recommendation and high quality of evidence [11]. In addition, SSRIs are recommended for overall symptom improvement in IBS patients though with a weak recommendation, and low quality of evidence. TCAs and SSRIs have effects on central pain and psychological distress and may also impact bowel function by improving diarrhea by slowing GI transit (TCAs), and ameliorating constipation by accelerating GI transit (SSRIs) [17, 18].

While we did not ask physicians if they were aware of any current risk scores for PI-IBS [19] or how using a risk score would change their current practice, the finding that the majority of physicians would use a risk score if available is promising. However previous studies have shown that PCPs are not aware of these tools and do not use them [6, 7]. This is understandable, given there is only one risk score currently published [19], and only one study that has used the score in a different patient population [20]. Future research in this area should focus on how existing or newly developed risk scores change current practice, diagnostic strategies, or patient outcomes for treatment of PI-IBS.

### Strengths & Limitations

This survey contributes to the literature about treatment and management of PI-IBS as no previous literature has focused on physician knowledge and treatment of PI-IBS. The small sample size is a noted limitation which likely affects the precision and generalizability of the estimates we described. Additionally we did not include nurse practitioners or physician assistants which are often “frontline” providers for treatment of IBS. Finally, after reviewing the wording of some of the questions in the survey, it is possible that some physicians responded to the questions given how they “should” approach a treatment or diagnostic step rather than what they actually do in their practice.

## Conclusion

These data need to be validated with larger systematic and representative surveys as well as direct patient surveys among those diagnosed with a high probability of PI-IBS. Furthermore, the current literature lacks estimates specifically for health utility/QALY impacts of PI-IBS specifically and these should be an immediate priority for future survey research.

## Data Availability

The datasets used and/or analysed during the current study are available from the corresponding author on reasonable request.

## Abbreviations

PCPs: Primary care physicians
PI-IBS: Post-infectious Irritable Bowel Syndrome
IBS: Irritable Bowel Syndrome
GPs: General practitioners
SD: Standard deviation
IQR: Interquartile range
QALY: Quality-Adjusted Life Years
TCAs: Tricyclic anti-depressants
SNRI: Serotonin or norepinephrine reuptake inhibitors
SSRI: Selective Serotonin Reuptake Inhibitor
